# Sparse evidence of MERS-CoV infection among animal workers living in Southern Saudi Arabia during 2012

**DOI:** 10.1111/irv.12287

**Published:** 2014-12-03

**Authors:** Ziad A Memish, Ahmad Alsahly, Malak al Masri, Gary L Heil, Benjamin D Anderson, Malik Peiris, Salah Uddin Khan, Gregory C Gray

**Affiliations:** a Ministry of HealthRiyadh, Saudi Arabia; b College of Medicine, Alfaisal UniversityRiyadh, KSA; c Regional Health DirectorateJazan, Saudi Arabia; d College of Public Health and Health Professions, Emerging Pathogens Institute, University of FloridaGainesville, FL, USA; e School of Public Health and the HKU-Pasteur Research Pole, The University of Hong KongHong Kong, Hong Kong; f Center for Communicable Diseases, ICDDR, BDhaka, Bangladesh

**Keywords:** antibody, Kingdom of Saudi Arabia, MERS-CoV, pseudoparticle virus neutralization assays

## Abstract

Middle East respiratory syndrome coronavirus (MERS-CoV) is an emerging viral pathogen that primarily causes respiratory illness. We conducted a seroprevalence study of banked human serum samples collected in 2012 from Southern Saudi Arabia. Sera from 300 animal workers (17% with daily camel exposure) and 50 non-animal-exposed controls were examined for serological evidence of MERS-CoV infection by a pseudoparticle MERS-CoV spike protein neutralization assay. None of the sera reproducibly neutralized the MERS-CoV-pseudotyped lentiviral vector. These data suggest that serological evidence of zoonotic transmission of MERS-CoV was not common among animal workers in Southern Saudi Arabia during July 2012.

## Introduction

First reported in the Kingdom of Saudi Arabia (KSA) in September 2012, Middle East respiratory syndrome coronavirus (MERS-CoV) is now frequently detected and causing high mortality among persons living or traveling in the Arabian Peninsula.^1^ Retrospective testing has revealed that MERS-CoV was also circulating in Zarqa, Jordan as early as April 2012, causing at least 2 deaths and likely 7 close contact infections.^2^ Since then, virus spread has seemingly increased. Between April 2012 and May 2014, there have been 536 reports of laboratory-confirmed human cases of MERS-CoV, of which, 145 (27%) have perished.^3^

The majority of the initially reported cases of MERS-CoV in KSA were sporadic, associated with severe illness, and diagnosed in tertiary care hospitals.^3^,^4^ As surveillance efforts have expanded, a number of MERS-CoV cases with milder clinical disease have been recognized. In this study of archive sera, we sought to examine evidence that subclinical MERS-CoV infections may have occurred in early 2012.

## Methods

### Study population

This study was approved by two institutional review boards (KSA Ministry of Health and Western IRB). Banked serum samples were collected using convenience sampling in July 2012 from 300 animal-exposed and 50 non-matched human controls residing in Jazan region, KSA, as part of a study investigating human Rift Valley fever exposure. The cross-sectional, convenience sample of human sera was chosen for its self-reported exposure to domestic animals, such as camels, as studies have documented increasing evidence that camels are a reservoir for MERS-CoV^5^,^6^ and human MERS-CoV infections were later reported in the area.

### Sample and data collection

After informed consent was obtained, demographic and domestic animal exposure data were collected using a structured questionnaire. Blood samples were then collected and transported on wet ice to the KSA Ministry of Health (MoH) laboratory in the Jazan region, KSA. Aliquots of sera were shipped to the University of Florida for serological assessment.

Questionnaire data were used to analyze demographic characteristics and generate animal exposure data. In addition, an epidemic curve was developed using published reports to identify the dates of onset for the MERS-CoV cases in KSA.^3^,^4^

### MERS-CoV spike pseudoparticle neutralization assay

A MERS-CoV spike protein pseudotyped lentiviral vector was constructed and provided by the laboratory of Malik Peiris of Hong Kong University. This assay is considered to be a more sensitive test compare to virus neutralization assay.^6^,^7^ The MERS-S pseudoparticles were used in a pseudoparticle neutralization test (ppNT) as first described elsewhere.^6^ In brief, the ppNT assay included infecting monolayers of Vero E6 cells (ATCC CRL-1586) in a single well (96-well plate format; 1 × 10^4^ cells/well) with HIVMERS-S-pseudotyped lentiviral vector having 5 ng p24. The MERS-S pseudoparticles were pre-incubated with serially diluted sera for 60 min at 4°C and then added to the cells in triplicate. After 2 days of incubation, 100 μl of luciferase substrate, ONE-Glo luciferase Assay System (Promega Madison, WI, USA), was added. The relative level of MERS-S pseudoparticle infection of the cells as luciferase activity was measured in a BioTek Synergy MX multimode plate reader (BioTek, Winooski, VT. Endpoint MERS-CoV neutralizing antibodies titers were determined as the highest serum dilution resulting in 90% reduction of luciferase relative to a negative serum (BSA) control. Polyclonal rabbit antisera to MERS-CoV obtained from the NIH/NIAID Rocky Mountain Research Laboratory was used as a positive control. Sensitivity of the assay in our laboratory was compared to that of the Peiris Laboratory at University of Hong Kong, where it was developed, by shipping an aliquot of this positive control which was independently titrated in the ppNT assay. Both our and Peiris' laboratory found the neutralizing titer of the polyclonal rabbit sera to be comparable to that measured by the Rocky Mountain Laboratories live virus neutralization assay (1:2560).

## Results

The study population was predominately male between 15 and 84 years of age, of them, approximately 84% of participants were between the ages of 20 and 59 years. The participants were enrolled from the governorates of Abu Arish, Al Aridah, Al Darb, Al Aydabi, Baish, Dhamad, Fifa, Jazan, Sabya, and Samtah. A small percentage (16.9%) of participants reported themselves to be semi-nomadic, with possible movement outside of the Jazan region. Nearly half (44%) of participants were from the Jazan Governorate. Among participants, 25% reported some form of contact with domestic animals including camels, cattle, horse, goats, or sheep and 17% reported daily contact with domestic camels during the 12 months to enrollment (see Table S1).

All serum samples were screened in triplicate at a dilution of 1:20. A specimen was considered as screening positive if the average of the three wells containing the sera exhibited a >50% reduction in luminescence in comparison to the luminescence negative (BSA) or no serum control. Thirteen sera (9 animal exposed and 4 controls) screened positive at 1:20, but upon titration of these sera in subsequent assays, none of them were reproducibly able to neutralize the MERS-CoV pseudotyped lentiviral vector at a serum dilution of 1:20.

**Figure 1:**
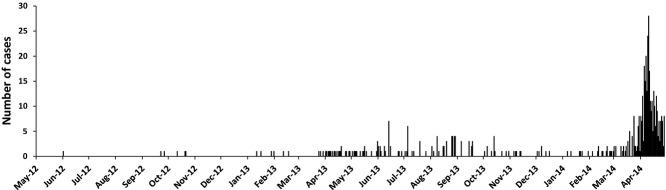
Epidemic curve of the MERS-CoV reported cases by date of onset, from May 1, 2012 to May 11, 2014 in the Kingdom of Saudi Arabia.

The epidemiological curve of human MERS-CoV cases in KSA (Figure1) reflects the occurrence of sporadic cases in 2012, low-level sustained transmission in 2013, and an epidemic spike between April and May 2014.

## Discussion

This study evaluated 350 banked serum samples collected in July 2012 from individuals with self-reported domestic animal exposure and controls in Jazan Region, KSA, for the presence of MERS-CoV neutralizing antibodies. None of the specimens tested positive using the MERS-S ppNT assay. Our negative results suggest population naivety to MERS-CoV infection in this region of KSA 1 month after the first earliest case was reported in another region of the country.^8^ At the time samples were collected, there was no known evidence of human or animal infections with MERS-CoV from the Jazan region.^4^ Since then multiple humans in Jazan have developed MERS-CoV infections.

As the first cases of MERS-CoV were identified, studies have postulated that camels are likely a reservoir for the virus in the Middle East.^5^,^6^,^9^,^10^ Given that 17% of participants of the animal-exposed participants reported daily contact with dromedary camels, we would have expected to find some serological evidence of infection if dromedary MERS-CoV was readily able to infect humans. Several other studies have reported similar results where no serological evidence of MERS-CoV infection was detected in domestic animal-exposed populations, including abattoir workers in sites where infected camels have been documented.^10^,^11^ During October 2012, researchers in KSA collected and tested serum samples from 226 abattoir workers in Jeddah and Makkah. Using an immunofluorescence assay, none had evidence of elevated MERS-CoV antibody.^11^ Similarly, in another 2013 study where nasal swabs from Egyptian camels suggested MERS-CoV was present, there was no evidence of seropositivity among the camel workers with intense contact to these same camels.^10^ These data are in parallel with the observed epidemiology of avian influenza H5N1 where serological, virological or clinical evidence of human infection is rare in spite of ubiquitous exposure. Perhaps, other mammalian species are serving as additional reservoirs for this pathogen or serving as vectors in transferring infection from camels to humans. Alternatively, perhaps our assay lacks sensitivity, or MERS-CoV infection may produce a weak or transient immune response following mild or asymptomatic infection. Finally, it is possible that only a subset of the human population has the predilection to be infected by MERS-CoV, either because of genetic polymorphisms or unusual routes of exposure.

Similar to MERS-CoV, severe acute respiratory syndrome (SARS) virus began causing sporadic self-limited outbreaks in late 2002, after which it seemingly developed sustained human-to-human transmission, and spread internationally.^12^ Post-epidemic studies of SARS have revealed a low-level circulation of the virus several months before it was first detected in humans in Guangdong Province of China, suggesting a period of time in which the virus adapted to human hosts.^13^ While it is difficult to draw direct comparisons, it seems possible that MERS-CoV is demonstrating a similar transmission pattern, reflected by the sporadic outbreaks occurring in 2012, followed by sustained low-level transmission in 2013, and now showing a larger human infection KSA wave in 2014 (Figure1).^4^ If the epidemiologic model of SARS is relevant to MERS,^13^ this current progression in MERS-CoV ecology may presage much greater future MERS-CoV epidemics.

There still remain many questions regarding designing appropriate interventions to prevent or reduce MERS-CoV transmission. Of them, identification of risk factors for MERS-CoV infection, a comprehensive understanding of natural animal reservoirs and vectors, and better determining transmission pathways seem to be paramount in developing appropriate interventions. Priority should be given to addressing these questions if we hope to improve public health response to future index cases in new geographical areas. It seems prudent to allocate resources to further globally enhance surveillance to quickly identify MERS-CoV and to monitor for viral genetic changes as well as to conduct the necessary epidemiological studies to fill the aforementioned gaps in our knowledge regarding this new and dangerous threat.

## Supporting Information

Table S1. Self-reported domestic animal exposure among participants, Jazan region of the Kingdom of Saudi Arabia, July 2012.
