# Supplementary material for: Sparse evidence of MERS-CoV infection among animal workers living in Southern Saudi Arabia during 2012
Source: Influenza Other Respir Viruses. 2014 Dec 3;9(2):64–7. doi: 10.1111/irv.12287 (PMC4353318; doi:10.1111/irv.12287)
Supplement: Supplementary file 1 [file irv0009-0064-sd1.docx]

| Table 1. Self-reported domestic animal exposure among participants, Jazan region of the Kingdom of Saudi Arabia, July 2012. | | | | | |
| --- | --- | --- | --- | --- | --- |
| **Animal type** | **Never**  **n (%)** | **Daily**  **n (%)** | **Weekly**  **n (%)** | **Monthly**  **n (%)** | **Any**  **n (%)** |
| Sheep | 54 (15%) | 272 (78%) | 8 (2%) | 16 (5%) | 296 (85%) |
| Goats | 67 (19%) | 258 (74%) | 9 (3%) | 16 (5%) | 283 (81%) |
| Cows | 217 (62%) | 106 (30%) | 14 (4%) | 12 (3%) | 132 (38%) |
| Camels | 259 (74%) | 61 (17%) | 15 (4%) | 13 (4%) | 89 (25%) |
| Horses | 330 (94%) | 4 (1%) | 2 (1%) | 11 (3%) | 17 (5%) |
| Dogs | 312 (89%) | 23 (7%) | 12 (3%) | 0 (0%) | 35 (10%) |
| Cats | 302 (86%) | 38 (2%) | 7 (2%) | 0 (0%) | 45 (13%) |
